# Synthesis of textured discontinuous-nanoisland Ca_3_Co_4_O_9_ thin films†

**DOI:** 10.1039/d2na00373b

**Published:** 2022-07-04

**Authors:** Binbin Xin, Arnaud Le Febvrier, Jun Lu, Biplab Paul, Per Eklund

**Affiliations:** Thin Film Physics Division, Department of Physics, Chemistry and Biology (IFM), Linköping University SE-58183 Linköping Sweden binbin.xin@liu.se per.eklund@liu.se

## Abstract

Controllable engineering of the nanoporosity in layered Ca_3_Co_4_O_9_ remains a challenge. Here, we show the synthesis of discontinuous films with islands of highly textured Ca_3_Co_4_O_9_, effectively constituting distributed nanoparticles with controlled porosity and morphology. These discontinuously dispersed textured Ca_3_Co_4_O_9_ nanoparticles may be a candidate for hybrid thermoelectrics.

## Abstract 2

Discontinuous films with islands of highly textured Ca_3_Co_4_O_9_ effectively constituting distributed nanoparticles can be obtained by controlling the Ca/Co ratio.

## Graphical abstract

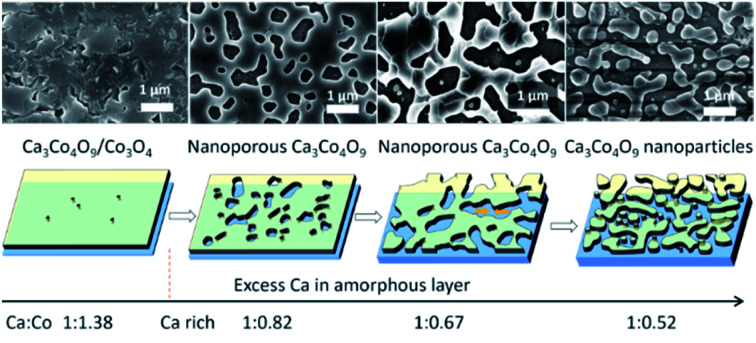

The misfit-layered calcium cobaltate Ca_3_Co_4_O_9_ has a complex crystal structure composed of CoO_2_ conductive layers and oxygen deficient rock-salt type Ca_2_CoO_3_ insulating layers.^1^ Ca_3_Co_4_O_9_ can be used in various energy-harvesting systems because of its high thermal stability and oxidation resistance. This material is an attractive p-type thermoelectric material with a high Seebeck coefficient *S*, moderate electrical conductivity *σ* and low thermal conductivity. It also can be used as an active material in Li-ion-battery anodes,^2,3^ hydrogen evolution and oxygen reduction reactions^4,5^ and supercapacitors with high cycling stability.^6,7^

Nanostructures such as nanoparticles^8^ and nanoporous films^9,10^ are common means to alter electrical, catalytic, and thermal properties of inorganic materials. In previous work, we have shown that nanoporous Ca_3_Co_4_O_9_ films on sapphire exhibit a thermal conductivity of 0.82 W m^−1^ K^−1^, which is nearly twofold lower than that obtained from comparable nonporous Ca_3_Co_4_O_9_ films.^11^ Furthermore, nanoporous Ca_3_Co_4_O_9_ films grown on mica can be obtained by reactions in hydrated CaO/CoO multilayers.^12^ The volume shrinkage in Ca(OH)_2_/Co_3_O_4_ multilayers and the out-of-plane orientation relationship between Ca(OH)_2_ and Co_3_O_4_ induce the formation of faceted and oriented nanopores in textured Ca_3_Co_4_O_9_ films.

Here, we show control of morphology and porosity in textured Ca_3_Co_4_O_9_ films, to form discontinuous films with islands of highly textured Ca_3_Co_4_O_9_, effectively constituting distributed nanoparticles. The discontinuous films with islands of highly textured Ca_3_Co_4_O_9_ were synthesized by radio-frequency (rf) sputtering followed by post-deposition annealing without any templates. Such films of discontinuously dispersed Ca_3_Co_4_O_9_ nanoparticles may be a promising filler in polymer matrixes for hybrid and composite materials in, *e.g.*, thermoelectrics.^13–16^

The Ca_3_Co_4_O_9_ nanoparticles were obtained by a similar method to that published in our earlier work.^12^ First, the CaO/Co_3_O_4_ multilayer films were deposited on muscovite mica (00l) and sapphire substrates (001) at 600 °C by reactive radio-frequency magnetron sputtering. The multilayers consisted of eight alternative bilayers of CaO (top layer) and Co_3_O_4_. The overall Ca : Co elemental ratio in the multilayer films was varied and set to 1 : 1.38 (close to stoichiometric Ca_3_Co_4_O_9_), 1 : 0.82, 1 : 0.67, and 1 : 0.52 by varying CaO and Co_3_O_4_ deposition times of their respective layer. Then, all the as-deposited multilayer films were exposed to a humid environment (0.88 relative humidity at constant temperature) to form Ca(OH)_2_/Co_3_O_4_ multilayer films at room temperature for two days, as described earlier.^12,17^ At the final stage, the different Ca(OH)_2_/Co_3_O_4_ multilayer films were annealed at 700 °C in air for 2 h.

X-ray diffraction (XRD) measurements were performed using an X'Pert PRO MRD diffractometer from PANalytical using Cu K_α1,2_ radiation with a nickel filter in the Bragg–Brentano configuration (*θ*–2*θ* scans). The surface morphology of the films was studied by scanning electron microscopy (SEM) using a LEO Gemini 1550 Zeiss with a 10 kV operating voltage. Transmission electron microscopy (TEM) was carried out on an FEI Tecnai G2 TF20 UT instrument operated at 200 kV. The Ca/Co elemental ratio was determined using energy-dispersive X-ray spectroscopy (EDS) by measuring at several positions on each sample growing on sapphire. The surface porosity fraction or coverage was determined from the SEM micrographs analysed using the software ImageJ (Java version).^18^ The electrical conductivity *σ* was calculated from the sheet resistance measured by using a four-point probe Jandel RM3000 station, and the film thickness was determined from the cross-sectional SEM images. The Seebeck coefficient was determined from the slope of the temperature gradient–voltage characteristics measured using a homemade Seebeck measurement setup system described elsewhere.^12,17^

Fig. 1 shows the X-ray diffraction patterns of the Ca_3_Co_4_O_9_ films on mica and sapphire as a function of the Ca/Co ratio: 1 : 1.38, 1 : 0.82, 1 : 0.67, and 1 : 0.52, respectively. The Ca/Co elemental ratios were measured from the different annealed films grown on sapphire by EDS and were the same as those in the as-deposited multilayer films grown on sapphire and mica and the annealed films on mica, respectively. Diffraction peaks for 001, 002, 003, 004, 005, and 006 reflections from Ca_3_Co_4_O_9_ and 111 and 222 reflections from Co_3_O_4_ can be observed in the film on mica with the initial composition (1 : 1.38, the closest to the stoichiometric Ca_3_Co_4_O_9_) in Fig. 1a. With decreasing Co content (Ca : Co 1 : 0.82 → 1 : 0.52), pure-phase Ca_3_Co_4_O_9_ can be identified in the films on mica from the XRD patterns (Fig. 1a). The intensity of peaks of Ca_3_Co_4_O_9_ growing on mica remains approximately the same in Fig. 1a. As known and observed from our earlier work,^19–21^ the excess Ca migrates and is incorporated in an amorphous layer between the nanoporous Ca_3_Co_4_O_9_ films and the mica substrate and will be discussed below. However, the pure-phase Ca_3_Co_4_O_9_ can be seen from the film growing on sapphire with Ca : Co = 1 : 1.38 (Fig. 1b). With increasing Ca content, additional CaO can be observed for the films on sapphire (Fig. 1b). This result indicates that some CaO remained in the annealed films on sapphire.

**Fig. 1:**
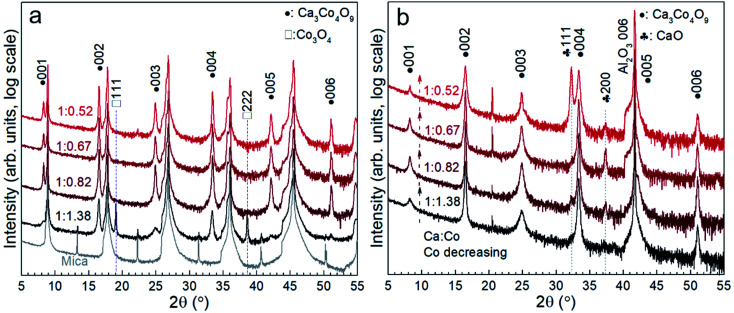
X-ray diffractograms of the annealed films grown on mica (a) and sapphire (b) with decreasing Co content in the films with Ca/Co ratios: 1 : 1.38, 1 : 0.82, 1 : 0.67, and 1 : 0.52, respectively.

The SEM images of the morphology of Ca_3_Co_4_O_9_ films on mica are shown in Fig. 2. The film on mica with the initial composition (1 : 1.38) shows morphology with few nanopores (Fig. 2a and e). With decreasing Co content, the morphology of Ca_3_Co_4_O_9_ in the annealed films change from a nanoporous continuous film morphology (Fig. 2b and f), *via* larger pores (Fig. 2c and g), to a discontinuous film of textured islands (Fig. 2d and h). The surface porosity fraction increases from 1.2% and 22% to 37% for the first three films (Table 1). For the discontinuous films, the corresponding value obtained from image analysis is an apparent “porosity” of 46% (Table 1), *i.e.* a surface coverage of 54%. This morphology is fundamentally different from the nanoporous films, though, in that the film is discontinuous and cannot be described as a porous film. The size of the nanoislands is mainly distributed from 50 nm to 1000 nm, as shown in Fig. S1.†

**Fig. 2:**
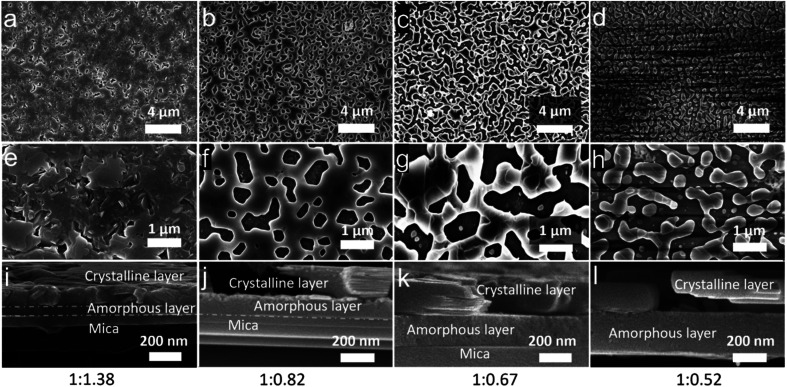
SEM images and cross-sectional SEM images for the annealed films grown on mica with the different ratios of Ca/Co: (a, e and i) 1 : 1.38; (b, f and j) 1 : 0.82; (c, g and k) 1 : 0.67; (d, h and l) 1 : 0.52.

**Table 1:** The amorphous layer apparent thickness, Ca_3_Co_4_O_9_ layer apparent thickness, and the apparent porosity fraction calculated from Fig. 2 as a function of the Ca : Co ratio in the films

Ca : Co elemental ratio	1 : 1.38	1 : 0.82	1 : 0.67	1 : 0.52
Thickness of the amorphous layer (nm)	81 ± 5	128 ± 6	179 ± 9	250 ± 12
Thickness of Ca_3_Co_4_O_9_ (nm)	121 ± 7	178 ± 9	194 ± 10	170 ± 9
Porosity (%)	1.2 ± 0.1	22 ± 1	37 ± 1.9	46 ± 2.3

The electrical conductivity and the Seebeck coefficient of the Ca_3_Co_4_O_9_/Co_3_O_4_ film are 27 S cm^−1^ and 139 μV K^−1^, respectively. The electrical conductivity of the nanoporous pure Ca_3_Co_4_O_9_ film decreases from 112 to 38 S cm^−1^ with increasing the porosity from 22% up to 37%. The Seebeck coefficient of the nanoporous Ca_3_Co_4_O_9_ films with different porosities is approximately 127 μV K^−1^, essentially the same for both. The Seebeck coefficient and electrical conductivity of the discontinuous film of textured Ca_3_Co_4_O_9_ islands cannot be measured by using these setups since there is no continuous conduction path.

The cross-sectional SEM micrographs (Fig. 2i–l) reveal that the films are composed of a crystalline layer on top of an amorphous layer. As is known from our earlier work,^20,21^ this amorphous layer forms due to a reaction between the mica substrate and the initial films during annealing. The amorphous layer contains O, Al, and Si elements from mica and Ca element from the initial films. The film growing on mica with the initial composition (1 : 1.38) shows a crystalline layer with a thickness of 121 nm and an amorphous layer with a thickness of 81 nm (Fig. 2i). This indicates that the formation of Ca_3_Co_4_O_9_ and amorphous layers occurs at same time during annealing. With increasing Ca content in the initial films, the pure crystalline Ca_3_Co_4_O_9_ layer of the last three films shows a similar apparent thickness of around 170 nm for all the films, but the thickness of the amorphous layer increases from 130 nm to 240 nm (Fig. 2j–l and Table 1).

The SEM images of the morphology of Ca_3_Co_4_O_9_ films on sapphire are shown in Fig. 3a–d. A dense film can be observed in the annealed film with Ca : Co = 1 : 1.38 (Fig. 3a). The nanoporous morphology with a nanopore size of ∼200 nm can be observed in the annealed film with low Ca : Co = 1 : 0.82 (Fig. 3b). Upon further decreasing the Co content, the surface morphology seems to be composed of a mixture of two families of grains (Fig. 3c). The similar round grains with a “size” (∼100 nm) can be observed on the top of the flat grains and nanopores at films 1 : 0.82 and 1 : 0.6 in Fig. 3b and c. For the lowest Co containing film, different family of grains can be observed forming a dense film (without apparent nanopores) (Fig. 3d).

**Fig. 3:**
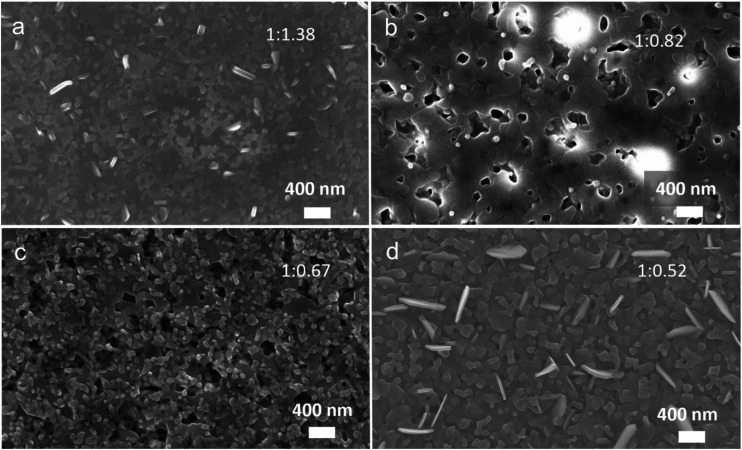
Surface SEM images for the annealed films grown on sapphire with the different ratios of Ca/Co: (a) 1 : 1.38; (b) 1 : 0.82; (c) 1 : 0.67; (d) 1 : 0.52.

The cross-sectional TEM images of the annealed film with the ratio of Ca : Co = 1 : 0.82 deposited on sapphire are shown in Fig. 4a and b. The nanopore structure in the Ca_3_Co_4_O_9_ layer with an apparent thickness of 185 nm can be observed in Fig. 4a, with the EDS maps of Co and Ca elements showing a uniform distribution in the Ca_3_Co_4_O_9_ layer but a higher Ca concentration in the nanopores. At the interface film substrate, a thin Ca_*x*_CoO_2_ layer can be seen near the sapphire substrate in Fig. 4b. The formation of Ca_*x*_CoO_2_ has been observed in earlier work.^22^ The lattice images for the Ca_3_Co_4_O_9_ layer and the SAED patterns (Fig. 4b) confirm that the (001) basal planes are oriented parallel to the film surface, corroborating the XRD results.

**Fig. 4:**
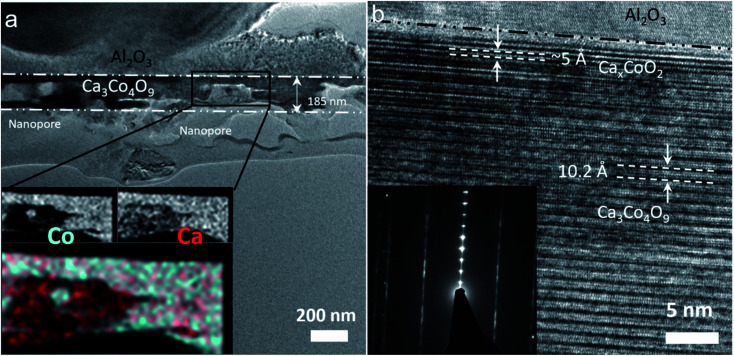
The cross-sectional TEM images of the annealed film with the ratio of Ca/Co = 1 : 0.82 deposited on sapphire: (a) low-magnification TEM image and the inset of EDS spectral maps of Ca and Co for nanopores, and (b) the corresponding high-resolution TEM images and inset of the SAED patterns capturing the layered atomic structure of Ca_3_Co_4_O_9_.

The dense Ca_3_Co_4_O_9_ film can be synthesized with the right Ca : Co elemental ratio (close to stoichiometric Ca_3_Co_4_O_9_) when the film grows on sapphire. The nanoporous film but a non-phase pure film mixing CaO and Ca_3_Co_4_O_9_ can form on sapphire with increasing Ca content. Comparing the results for the films grown on sapphire with those on mica allows determining the mechanism of the increase in the porosity fraction and formation of a discontinuous film of islands, effectively constituting distributed nanoparticles.

This discontinuous structure is correlated with the reaction between Ca in the Ca(OH)_2_/Co_3_O_4_ multilayer films with the mica layer. In our previous work, pore formation could be attributed to the basal plane removal driven by local densification of textured Ca_3_Co_4_O_9_ nuclei during growth.^12^ In the present case, this mechanism yields formation of a discontinuous film of islands, *i.e.*, distributed nanoparticles, for the high initial Ca content in the starting multilayers. A schematic illustration is shown in Fig. 5. When Ca : Co = 1 : 1.38 (close to 3 : 4), the film with few nanopores is composed of a crystalline Ca_3_Co_4_O_9_/Co_3_O_4_ layer on top of a thin amorphous layer, which proves that Ca diffuses and reacts with the mica substrate to form an amorphous layer during formation of Ca_3_Co_4_O_9_. During annealing and with increased Ca content, the excess Ca from Ca(OH)_2_ will be attracted to the interface substrate/film where the reaction occurs to form a thicker amorphous layer underneath phase pure crystalline Ca_3_Co_4_O_9_ layers with nanopores. With further increase of Ca content, the nanopore size and porosity significantly increase, while the apparent thickness of the crystalline Ca_3_Co_4_O_9_ layer remains constant. This result indicates that the volume shrinkage of Ca_3_Co_4_O_9_ preferentially occurs in the in-plane direction and not in the out-of-plane direction. As expected, the more excess Ca results in a thicker amorphous layer with even lower Co content. Instead of forming nanoporous Ca_3_Co_4_O_9_, the Ca_3_Co_4_O_9_ instead forms a discontinuous film of islands, constituting distributed nanoparticles with a larger apparent “porosity”.

**Fig. 5:**
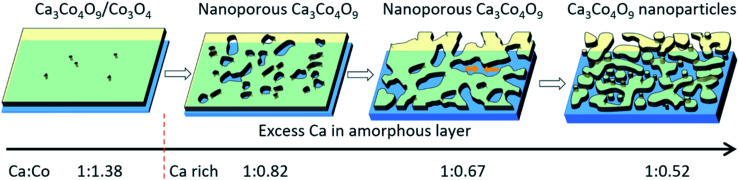
Schematic illustration of formation of Ca_3_Co_4_O_9_ nanoparticles.

The growth of discontinuous films with islands of highly textured Ca_3_Co_4_O_9_ effectively constituting distributed nanoparticles has been demonstrated by sequential sputtering-annealing without any templates. The volume shrinkage in the initial Ca(OH)_2_/Co_3_O_4_ multilayers with different Ca/Co overall ratios can be used to tailor morphology and surface coverage porosity in textured Ca_3_Co_4_O_9_ films. Such films of discontinuously dispersed Ca_3_Co_4_O_9_ nanoparticles may be a promising filler in polymer matrixes for hybrid and composite materials in, *e.g.*, hybrid thermoelectrics.

## Conflicts of interest

There are no conflicts to declare.

## Supplementary Material

NA-004-D2NA00373B-s001

## References

[cit1] Miyazaki Y., Onoda M., Oku T., Kikuchi M., Ishii Y., Ono Y., Morii Y., Kajitani T. (2002). J. Phys. Soc. Jpn..

[cit2] Kim D. W., Ko Y. D., Park J. G., Kim B. K. (2007). Angew. Chem., Int. Ed..

[cit3] Guan S., Fan Q., Liu L., Luo J., Zhong Y., Zhao W., Huang Z., Shi Z. (2020). Sci. China: Technol. Sci..

[cit4] Lim C. S., Chua C. K., Sofer Z., Jankovský O., Pumera M. (2014). Chem. Mater..

[cit5] Silva V. D., Simões T. A., Loureiro F. J. A., Fagg D. P., Medeiros E. S., Macedo D. A. (2018). Mater. Lett..

[cit6] Wang Z., Wang Y., Yue X., Shi G., Shang M., Zhang Y., Lv Z., Ao G. (2019). J. Alloys Compd..

[cit7] Mendoza R., Oliva J., Padmasree K. P., Oliva A. I., Mtz-Enriquez A. I., Zakhidov A. (2022). J. Energy Storage.

[cit8] Snyder J., McCue I., Livi K., Erlebacher J. (2012). J. Am. Chem. Soc..

[cit9] Guo D.-J., Ding Y. (2012). Electroanalysis.

[cit10] Tang J., Wang H. T., Lee D. H., Fardy M., Huo Z., Russell T. P., Yang P. (2010). Nano Lett..

[cit11] Paul B., Zhang Y., Zhu W., Xin B., Ramanath G., Borca-Tasciuc T., Eklund P. (2022). Appl. Phys. Lett..

[cit12] Xin B., Febvrier A. L., Shu R., Elsukova A., Venkataramani V., Shi Y., Ramanath G., Paul B., Eklund P. (2021). ACS Appl. Nano Mater..

[cit13] Du Y., Xu J., Paul B., Eklund P. (2018). Appl. Mater. Today.

[cit14] Wang Y., Yang L., Shi X. L., Shi X., Chen L., Dargusch M. S., Zou J., Chen Z. G. (2019). Adv. Mater..

[cit15] Zhang L., Shi X.-L., Yang Y.-L., Chen Z.-G. (2021). Mater. Today.

[cit16] Wang L., Zhang Z., Liu Y., Wang B., Fang L., Qiu J., Zhang K., Wang S. (2018). Nat. Commun..

[cit17] Xin B., Febvrier A. L., Wang L., Solin N., Paul B., Eklund P. (2021). Mater. Des..

[cit18] Abràmoff M. D., Magalhães P. J., Ram S. J. (2004). Biophot. Int..

[cit19] Paul B., Lu J., Eklund P. (2017). ACS Appl. Mater. Interfaces.

[cit20] Paul B., Björk E. M., Kumar A., Lu J., Eklund P. (2018). ACS Appl. Energy Mater..

[cit21] Xin B., Ekström E., Shih Y.-T., Huang L., Lu J., Elsukova A., Zhang Y., Zhu W., Borca-Tasciuc T., Ramanath G., Le Febvrier A., Paul B., Eklund P. (2022). Nanoscale Adv..

[cit22] Paul B., Schroeder J. L., Kerdsongpanya S., Nong N. V., Schell N., Ostach D., Lu J., Birch J., Eklund P. (2015). Adv. Electron. Mater..

