# Supplementary material for: Synthesis of textured discontinuous-nanoisland Ca3Co4O9 thin films
Source: Nanoscale Adv. 2022 Jul 4;4(16):3318–22. doi: 10.1039/d2na00373b (PMC9416933; doi:10.1039/d2na00373b)
Supplement: NA-004-D2NA00373B-s001 [file NA-004-D2NA00373B-s001.pdf]

Electronic Supplementary Material (ESI) for Nanoscale Advances.
This journal is © The Royal Society of Chemistry 2022

Supporting Information

Synthesis of textured discontinuous-nanoisland $Ca_3Co_4O_9$ thin films

*Binbin Xin[a, *], Arnaud Le Febvrier[a], Jun Lu[a], Biplab Paul,[a,b], and Per Eklund[a, *]*

a, Thin Film Physics Division, Department of Physics, Chemistry and Biology (IFM),

Linköping University, SE-58183 Linköping, Sweden

b: Present address: Platit AG, Selzach, Switzerland

*Corresponding authors. binbin.xin@liu.se; per.eklund@liu.se

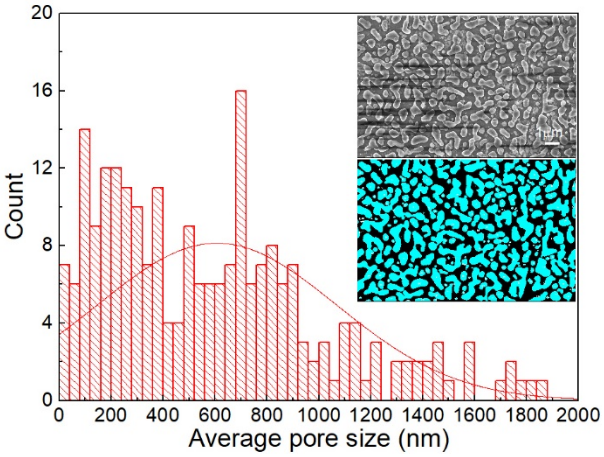

Figure S1 Nanoisland size distribution of discontinuous films with islands of highly textured $Ca_3Co_4O_9$. The inset images are the SEM image of the film grown on mica with the Ca/Co ratio (1:0.52) and image treated by ImageJ from the SEM image.

The shape of nano islands is irregular, but most of the nano islands are close hexagonal in shape. The average nano island sizes are estimated from the nanopore area with an uncertainty of 30%, by assuming all the pores to be regular hexagons. The nano islands area is calculated from analyses of SEM images by ImageJ software. The average nano island size is equal to L+ 2Lcos 60° where L is hexagon side length.
